# Return to Play and Performance After Anterior Cruciate Ligament Reconstruction in Soccer Players: A Systematic Review of Recent Evidence

**DOI:** 10.1007/s40279-024-02035-y

**Published:** 2024-05-06

**Authors:** Marko Manojlovic, Srdjan Ninkovic, Radenko Matic, Sime Versic, Toni Modric, Damir Sekulic, Patrik Drid

**Affiliations:** 1 Faculty of Sport and Physical Education, University of Novi Sad, Novi Sad, Serbia; 2 Faculty of Medicine, University of Novi Sad, Novi Sad, Serbia; 3 Department of Orthopedic Surgery and Traumatology, Clinical Center of Vojvodina, Novi Sad, Serbia; 4 Faculty of Kinesiology, University of Split, Split, Croatia

## Abstract

**Background:**

The available literature referring to the return to play (RTP) and performance after anterior cruciate ligament reconstruction (ACLR) has already been comprehensively summarized in team sports such as basketball or American Football. Therefore, in this sense, it is necessary to synthesize evidence relating to the mentioned parameters in soccer players who underwent ACLR.

**Objective:**

The aim of this systematic review was to examine RTP and the performance of soccer players after ACLR.

**Methods:**

Three electronic databases, Web of Science, Scopus, and PubMed, have been comprehensively searched to identify relevant articles. The following inclusion criteria were applied: (1) the sample of respondents consisted of soccer players irrespective of their age, sex, or level of competition; (2) athletes experienced anterior cruciate ligament injury and underwent ACLR; (3) outcomes estimated referred to the RTP, RTP at the preinjury level of competition, RTP time, performance, and career duration of soccer players; (4) studies were written in the English language. The methodological quality of the research was evaluated using the Methodological Index for Non-Randomized Studies (MINORS).

**Results:**

Databases searched yielded a total of 694 studies, of which 17 fulfilled the eligibility criteria and were included in the final analysis. These included 3657 soccer players, 2845 males and 812 females, who underwent ACLR and most commonly competed at the elite, national, amateur, and recreational levels. The results obtained indicated that 72% of soccer players successfully RTP and 53% RTP at the preinjury level of participation after ACLR. In addition, recent evidence provided in this literature review demonstrated that mean RTP time was 264 days or 8.7 months. Moreover, the majority of the studies unambiguously suggested that performance related to statistical aspects noticeably deteriorated compared with both the preinjury period and noninjured athletes. The mean career length of soccer players following ACL surgery was approximately between 4 and 5 years.

**Conclusion:**

Although a high percentage of athletes RTP after a relatively short period of absence from the sports field compared with other sports closely related to soccer, ACLR negatively impacts soccer players’ performance and career duration.

**Supplementary Information:**

The online version contains supplementary material available at 10.1007/s40279-024-02035-y.

## Key Points

**Table:** 

The main findings were that 72% of soccer players return to play (RTP) after anterior cruciate ligament reconstruction (ACLR), while 53% RTP at the preinjury level of competition.
The literature reported that mean RTP time following ACLR was approximately 264 days or 8.7 months, including all levels of play.
There is quite firm scientific evidence that the performance and career length of soccer players significantly deteriorated after surgery of the anterior cruciate ligament.

## Introduction

According to the scientific literature, soccer represents one of the most popular sports in the world [1, 2]. To be successful in soccer, it is necessary to continually develop physiological, psychological, technical, and tactical performance [1]. Moreover, the risk of injuries has also grown due to the increased physical demands of soccer over the last several decades. It is noteworthy to highlight that injuries in soccer players most commonly affect knee and ankle joints and muscles of the thigh and calf [3].

Anterior cruciate ligament (ACL) rupture is one of the most explored phenomena in sports science and medicine [4]. ACL is considered the vital structure of the knee joint due to its crucial role in stabilization and kinematics [5, 6]. ACL injuries are very common in soccer, and they can induce substantial impairments in athletes’ quality of life, as well as enormous financial costs for society [7]. Regarding risk factors for ACL rupture, female sex and older age markedly increase the frequency of ACL injuries [8]. Furthermore, numerous studies have investigated the incidence of ACL injuries in soccer players [9–11]. For instance, Grassi et al. [9] revealed that the incidence of ACL rupture during matches was 0.4215/1000 h in elite Italian soccer athletes competing in Serie A. In addition, ACL injuries have been far more prevalent in amateur German soccer players than in professional and semi-professional athletes [11]. Several studies have also emphasized that most of the soccer players that experienced ACL rupture underwent ACL reconstruction (ACLR) [12, 13].

Indeed, there is abundant evidence concerning return to play (RTP) in athletes after ACLR [14–17]. More precisely, Ardern et al. [15] performed a systematic review and meta-analysis that included 45 studies with 5770 participants. The authors reported that 82% of respondents successfully RTP following ACLR. Moreover, fear of reinjury was the most stated reason for the decrease or complete cessation of sport participation. Of note, the available literature also indicates that 53% [16] or even 65% [17] of athletes RTP at the preinjury level of competition after ACLR. A period of 6–13 months was necessary to recover and return to the sports field [18]. Overall, based on the highlighted facts, it is apparent that there is compelling evidence that a truly high percentage of athletes can successfully recuperate and return to sport following surgery of the ACL.

In terms of performance, the scientific literature is quite equivocal. Several investigations demonstrated significant deterioration of performance after ACLR [19–21], while in other studies, differences were not revealed [22, 23]. For example, Read et al. [20] examined sport-specific performance in National Football League (NFL) (American Football League) defensive players after ACLR. The obtained results exhibited a decrease in performance, including started games and solo tackles per game, in the ACLR group of athletes, while no changes were observed in the control group. Similarly, a noticeable decline in the performance of National Basketball Association (NBA) players, such as games played per season, minutes, points, and rebounds per game, was found in the season following ACL surgery compared with the preinjury period [21]. Additionally, it was emphasized that the mean career length of NBA players after ACLR was approximately 4.3 years. In contrast, no deterioration of performance variables was noted in hockey players that underwent ACLR [23].

To date, to the best of the authors’ knowledge, several systematic reviews have been conducted regarding RTP and performance after ACLR in team sports, such as basketball [24] and American Football [25]. In this sense, there is an obvious need to extend and deepen the current body of knowledge referring to the mentioned parameters in soccer players that underwent reconstruction of the ACL. Therefore, the objective of the presented research was to summarize recent evidence relating to the RTP and sport-specific performance in soccer players following ACLR.

## Methods

### Search Strategy

In order to provide innovative evidence referring to the return to soccer and performance after ACLR, Web of Science, Scopus, and PubMed were comprehensively searched from January 1st, 2018, to April 25th, 2023 (literature available within the last 5 years). A Boolean search syntax was applied using the operators ‘AND’ and ‘OR’ with the following keywords: (‘return to play’ OR ‘return to play at the preinjury level of participation’ OR ‘return to play time’) AND (‘performance’ OR ‘sport-specific performance’) AND (‘career length’ OR ‘career duration’) AND (‘anterior cruciate ligament’ OR ‘anterior cruciate ligament reconstruction’ OR ‘ACL’ OR ‘ACL injury’) AND (‘soccer’ OR ‘football’ OR ‘athletes’). Regarding the search for other sources, reference lists of all the relevant articles were thoroughly checked to identify additional studies. One independent reviewer (MM) performed searches of three electronic databases to find records eligible for inclusion. Selection of the retrieved trials, including screening of titles and abstracts, as well as analysis of full-text articles, was carried out independently by two reviewers (SV and TM). Any potential disagreements between reviewers were clarified through discussion or after a meeting and consultation with the first author (MM). This systematic review of the recent literature was conducted according to the guidelines of the Preferred Reporting Items for Systematic Review and Meta-Analyses (PRISMA) statement [26]. The study protocol has been registered in the International Prospective Register of Systematic Reviews (PROSPERO) with the reference number CRD42023417745.

### Eligibility Criteria

To be included in the presented literature review, the studies needed to meet the following eligibility criteria: (1) the sample consisted of soccer players irrespective of their age, sex, or level of competition; (2) the population involved experienced ACL injury and underwent ACLR; (3) outcomes assessed pertained to the RTP, RTP at the preinjury level of competition, RTP time, performance, or career length of soccer players; (4) written in the English language. Research was excluded if (1) the examined population comprised soccer players and athletes competing in other sports; (2) multi-ligament knee injuries or surgeries were recorded; (3) American Football players were included. Finally, non-peer-reviewed journal articles, case reports, conference papers, editorials, systematic reviews, and meta-analyses were also not considered for inclusion in this investigation.

### Data Extraction and Synthesis

Two independent reviewers (SN and RM) conducted data extraction, and inconsistencies were resolved by consensus. Thereafter, retrieved data from each of the studies were entered into a Microsoft Excel template. Extracted data included name of the first author and year of publication, design of the included studies, and the level of evidence. In terms of characteristics of participants, data such as sample size, mean age, sex, country of soccer players, competitive level, and type of ACL surgery were retrieved from all the investigations. Of note, information relating to the graft type used was also extracted into Microsoft Excel. Concerning relevant outcomes, data pertaining to the RTP, RTP at the preinjury level of participation, RTP time after ACLR, and reasons for not returning to soccer were included. In addition, data regarding the performance and career duration of soccer athletes were retrieved and presented in the manuscript.

Due to the high level of heterogeneity among studies with respect to their design, population characteristics, and particularly outcomes estimated, meta-analysis was not a suitable option. Most importantly, considerable heterogeneity was observed in terms of levels of play. The available literature addressed elite, national-level, amateur, recreational, and youth soccer players. Thus, the results reported in each study were synthesized and presented descriptively.

### Definitions of Return to Play (RTP), Performance, and Career Length

For the purpose of this systematic review, RTP was defined as the number or percentage of soccer players that were able to play in at least one competitive game following ACLR. RTP at the preinjury level represented the number or percentage of athletes that were able to play soccer at the same competitive level as in the season before ACLR. RTP time was defined as the number of days or months from ACLR to the first competitive soccer match appearance. Furthermore, performance refers to the statistical aspects of soccer, including games and minutes played per season or parameters recorded during soccer matches such as scored goals, assists, dribblings, passes, etc. Career length represented the number of years or seasons in which athletes successfully played soccer after ACLR.

### Quality Assessment

The evaluation of the methodological quality of the articles involved in this literature review was carried out using the Methodological Index for Non-Randomized Studies (MINORS) [27]. MINORS assesses eight aspects of noncomparative studies: a clearly stated aim, the inclusion of respondents, data collection, estimation of the outcomes, blind evaluation of the endpoints, the length of the follow-up period, loss to follow-up, and calculation of the sample size necessary for the study. For comparative studies, the following four additional criteria are rated: an appropriate description of a control group, contemporary groups, baseline comparisons of groups, and statistical analysis. All items are evaluated with a score of 0 (not reported), 1 (reported but inadequate), or 2 (reported and adequate); hence, the maximum final scores are 16 and 24 for noncomparative and comparative studies, respectively. In the investigations without a control group, the quality score was interpreted as follows: 0–4 very low; 5–8 low; 9–12 moderate; 13–16 high [28]. Conversely, in studies with a control group, the final quality score was categorized as follows: 0–6 very low; 7–12 low; 13–18 moderate; 19–24 high [28]. The quality assessment of the studies was performed by two independent reviewers (SV and TM). All discrepancies were resolved following consultation with the first author (MM).

## Results

### Search Results and Study Characteristics

**Fig. 1:**
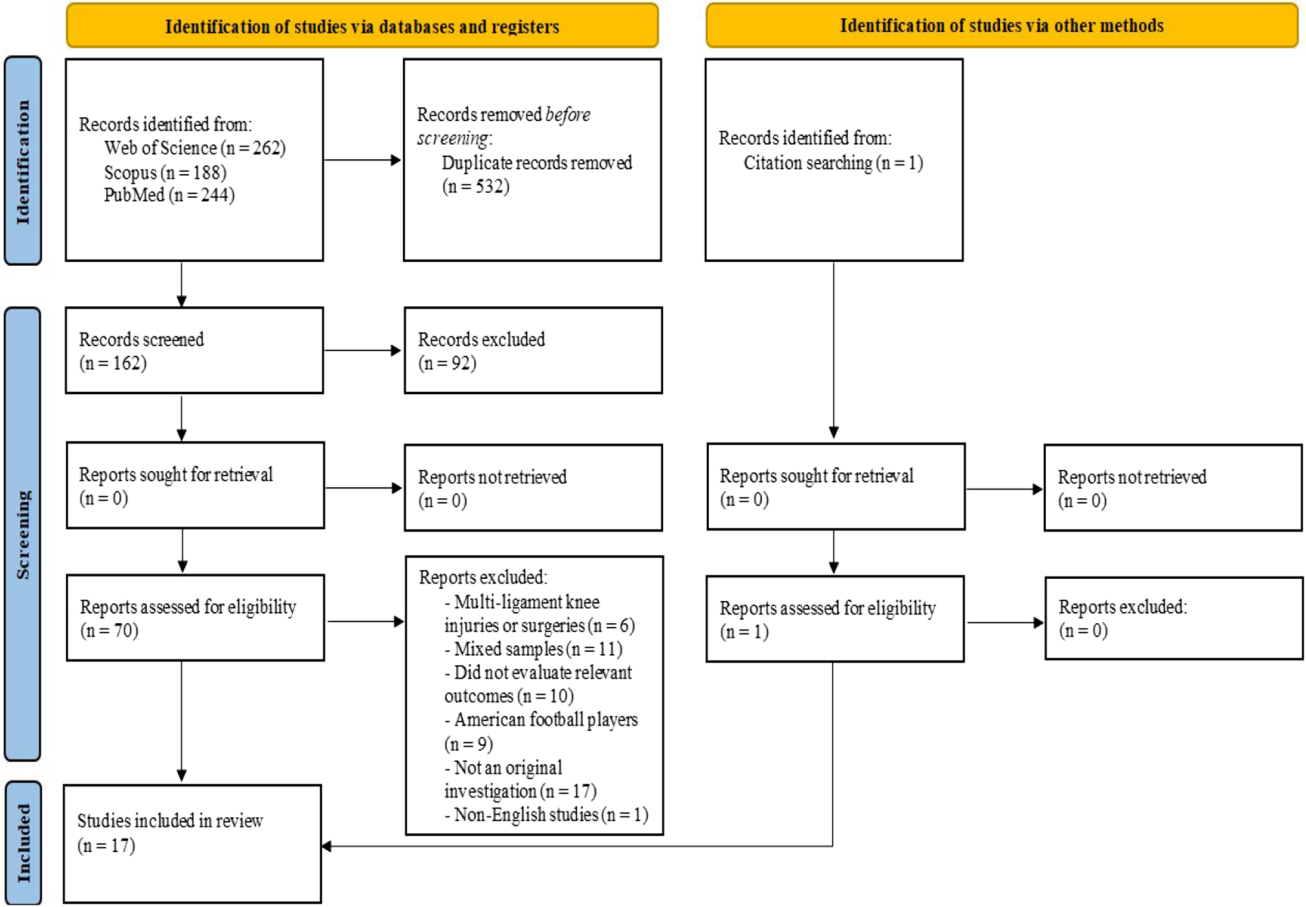
PRISMA flow diagram illustrates the search strategy

Figure 1 illustrates the complete results concerning the study selection process. Firstly, a comprehensive search of all the databases yielded a total of 694 records, with one additional record identified via reference citation checking. Secondly, after eliminating duplicates, the titles and abstracts of 162 papers were screened. Thirdly, 92 trials were removed, and 70 full-text articles were assessed for eligibility. Finally, 54 reports were excluded with reasons, and 17 studies (inclusive of the study identified by reference citation checking) were included in the presented systematic review of recently available scientific evidence.

In terms of study design, there were six cohort studies [30, 32, 38, 40, 44, 45], five case series [29, 31, 33, 34, 37], three case–control studies [39, 42, 43], two prospective cohort studies [35, 36], and one descriptive epidemiology study [41], and level II [35, 36], level III [30, 32, 38–45], and level IV [29, 31, 33, 34, 37] of evidence were recorded. Level II evidence refers to prospective cohort studies, level III of evidence refers to case–control and cohort studies, while level IV evidence refers to case series. A total of 3657 soccer players, 2845 males and 812 females, with the mean age ranging from 15.4 to 30 years, underwent ACLR in studies involved in the final analysis. Athletes most commonly competed in the prestigious European soccer leagues, including England, France, Germany, Italy, Spain, Portugal, and Belgium, but also in the United States, Australia, and Chile. Ten [30–33, 35, 37, 38, 41, 42, 44] out of 17 investigations examined elite soccer players exclusively, whilst there were also national-level [36, 43], amateur [29, 45], recreational [40, 43], and youth athletes [36, 40, 43]. Concerning ACLR, in nine studies, soccer players underwent both primary and revision of ACLR [32, 33, 35, 36, 38, 42–45]. In seven studies, they experienced only primary ACLR [29–31, 34, 37, 39, 41], and one study provided evidence relating to the revision of ACLR [40]. Of the available graft types shown in Table 1, hamstring autografts, bone-patellar tendon-bone autografts, patellar tendon autografts, quadriceps tendon autografts, and allografts were used in the majority of the studies.

**Table 1:** Study design and sample characteristics

Study	Study design (level of evidence)	Sample size, *n*	Mean age, y	Sex	Country (competitive level of athletes)	ACLR (primary or revision)	Graft type
Alonso et al. (2019) [29]	Case series (IV)	61	29.7 ± 5.5	Male	Chile (amateur soccer players)	Primary	Hamstring autograft and BTB
Arundale et al. (2018) [30]	Cohort (III)	54	24.9 ± 3.8	Male	United States (MLS elite soccer players)	Primary	BTB and hamstring autograft
Balendra et al. (2022) [31]	Case series (IV)	232	23.3 ± 4.4	Male = 205 Female = 27	United Kingdom (elite soccer players)	Primary	PT, hamstring graft, and allograft
Barth et al. (2019) [32]	Cohort (III)	176	26.1 ± 3.8	Male	England, France, Germany, Italy, Spain, and the United States (elite soccer players)	Primary and revision	NA
Bonanzinga et al. (2022) [33]	Case series (IV)	28	25.3 ± 5.0	Male	Italy (elite soccer players)	Primary and revision	Hamstring graft
Britt et al. (2020) [34]	Case series (IV)	71	15.4 ± 1.3	Female	United States (NA)	Primary	Hamstring autograft and BTB
Della Villa et al. (2021) [35]	Prospective cohort (II)	118	25.0 ± 4.3	Male	NA (elite UEFA soccer players)	Primary and revision	PT, hamstring autograft, iliotibial autograft, QT, and different allografts
Fältström et al. (2021) [36]	Prospective cohort (II)	222	18.4 ± 2.7	Female	Sweden (elite, national-level, and youth soccer players)	Primary and revision	Hamstring autograft, PT, and QT
Farinelli et al. (2023) [37]	Case series (IV)	27	23.2 ± 4.3	Male	England, Germany, and Italy (elite UEFA soccer players)	Primary	BTB and QT
Forsythe et al. (2021) [38]	Cohort (III)	51	24.9 ± 4.1	Male	England, France, Germany, Italy, and Spain elite UEFA soccer players)	Primary and revision	NA
Manara et al. (2022) [39]	Case–control (III)	862	30.0 ± 4.2	Male = 666 Female = 196	Australia (NA)	Primary	Hamstring autograft
Mars Group (2021) [40]	Cohort (III)	90	24.0 ± 8.5	Male = 46; Female = 44	NA (recreational and youth soccer players)	Revision	BTB, hamstring autograft, and allograft
Mazza et al. (2022) [41]	Descriptive epidemiology (III)	183	25.4 ± 3.9	Male	Belgium, England, France, Germany, Italy, Portugal, and Spain (elite UEFA soccer players)	Primary	NA
Niederer et al. (2018) [42]	Case–control (III)	125	25.3 ± 4.2	Male	England, France, Germany, Italy, and Spain (elite soccer players)	Primary and revision	NA
Sandon et al. (2019) [43]	Retrospective case–control (III)	684	25.9 ± 8.6	Male = 432 Female = 252	Sweden (national-level, recreational, and youth soccer players)	Primary and revision	BTB and hamstring graft
Schiffner et al. (2018) [44]	Retrospective cohort (III)	66	24.0 ± 3.6	Male	Germany (elite soccer players)	Primary and revision	NA
Szymski et al. (2023) [45]	Cohort (III)	607	24.7 ± 4.1	Male	Germany (elite, semi-professional, and amateur soccer players)	Primary and revision	NA

*ACLR* anterior cruciate ligament reconstruction, *BTB* bone-patellar tendon-bone, *MLS* Major League Soccer, *NA* not applicable, *PT* patellar tendon, *QT* quadriceps tendon, *UEFA* Union of European Football Associations

### RTP After Anterior Cruciate Ligament Reconstruction (ACLR) in Soccer Players

Twelve studies [30–34, 36, 37, 39–43] explored RTP in soccer players following ACLR. Overall, 72% (1981/2735) of athletes had been able to RTP after ACL surgery. Regarding RTP at the preinjury level, taking into account data provided in 11 [29–31, 33–35, 37, 38, 40, 42, 43] out of 17 articles, 53% (783/1486) of soccer athletes successfully RTP at the same level of participation as before ACLR. More details referring to the RTP and RTP at the preinjury level of competition are provided in Table 2.

**Table 2:** RTP after ACLR in soccer players

Study	RTP, *n* (%)	RTP at the preinjury level, *n* (%)	RTP time, days (months)	Reasons for not returning to soccer
Alonso et al. (2019) [29]	NA	30/61 (49)	NA	Fear of reinjury; knee-related issues and lack of knee confidence; family or job commitments; not eligible to participate in competitive sport
Arundale et al. (2018) [30]	40/54 (74)	37/54 (69)	NA	NA
Balendra et al. (2022) [31]	222/231 (96)	209/231 (90)	319 (10.5)	NA
Barth et al. (2019) [32]	164/176 (93)	NA	311 (10.2)	NA
Bonanzinga et al. (2022) [33]	27/28 (97)	23/28 (82)	243 (8.0)	NA
Britt et al. (2020) [34]	47/71 (66)	26/71 (37)	NA	Fear of reinjury; decreased condition; pain; busy
Della Villa et al. (2021) [35]	NA	49/82 (60)	224 (7.4)	NA
Fältström et al. (2021) [36]	163/222 (73)	NA	NA	NA
Farinelli et al. (2023) [37]	25/27 (93)	23/27 (85)	256 (8.4)	NA
Forsythe et al. (2021) [38]	NA	41/51 (80)	216 (7.1)	NA
Manara et al. (2022) [39]	602/862 (70)	NA	NA	Operated knee; fear of reinjury
Mars Group (2021) [40]	45/72 (63)	35/72 (49)	292 (9.6)	Knee-related issues
Mazza et al. (2022) [41]	174/183 (95)	NA	248 (8.2)	NA
Niederer et al. (2018) [42]	123/125 (98)	75/125 (60)	210 (6.9)	NA
Sandon et al. (2020) [43]	349/684 (51)	235/684 (34)	NA	Knee-related issues including pain and instability; fear of reinjury
Schiffner et al. (2018) [44]	NA	NA	244 (8.0)	NA
Szymski et al. (2023) [45]	NA	NA	337 (11.1)	NA

*ACLR* anterior cruciate ligament reconstruction, *NA* not applicable, *RTP* return to play

RTP time was evaluated in 11 investigations [31–33, 35, 37, 38, 40–42, 44, 45]. The average RTP time following ACL surgery was 264 days or 8.7 months (Table 2). Interestingly, one study [40] examined RTP time in recreational and amateur soccer players undergoing revision of ACLR. Athletes returned to the soccer field after 292 days or 9.6 months. Moreover, Szymski et al. [45] showed that RTP time in elite, semi-professional, and amateur athletes with a history of primary and revision of ACLR was 337 days or 11.1 months. Further, five studies [29, 34, 39, 40, 43] addressed reasons why athletes did not return to soccer after ACLR. Most importantly, knee-related issues [29, 39, 40, 43] and fear of reinjury [29, 34, 39, 43] were most commonly cited as reasons for not returning to soccer. Family and job commitments [29], reduced physical condition, and lack of time [34] were also specified as reasons for athletes being unable to return to soccer after ACLR.

### Performance and Career Length After ACLR in Soccer Players

Soccer-specific performance was reported in seven studies [30, 32, 37, 38, 41, 42, 45], which evaluated various statistical parameters including minutes played per game, minutes played per season, games played per season, achieved goals or assists, number of successful dribblings, etc. (Table 3). Two studies [32, 37] provided inconsistent results concerning comparison within the group; several variables deteriorated relative to the preinjury period, while some remained unchanged. Conversely, four studies [38, 41, 42, 45] unambiguously demonstrated a substantial decrease in assessed performance after ACLR compared with the season before surgery. Likewise, compared with a control group, a significant decline in performance was observed in two studies [30, 42]. Only Forsythe et al. [38] did not find differences between athletes who underwent ACLR and their healthy counterparts.

**Table 3:** Performance and career length after ACLR in soccer players

Study	Performance (comparison within the group)	Performance (comparison with a control group)	Career length	Career length (comparison with a control group)
Arundale et al. (2018) [30]	NA	ACLR group had a significantly lower percentage of regular or postseason games started and a significantly higher percentage of regular/postseason games that they did not play compared with control group athletes	1.3 years after ACLR	ACLR group had significantly shorter soccer careers relative to the control group
Barth et al. (2019) [32]	GPS, MPS, MPG, and GLPS substantially decreased in the seasons following ACLR relative to the preinjury period. Conversely, there were no differences in parameters such as SPS, SGPS, APS, GCPS, or yellow and red cards per season	NA	4 seasons after ACLR	NA
Della Villa et al. (2021) [35]	NA	NA	4.1 years after ACLR	NA
Farinelli et al. (2023) [37]	A statistically significant decrease in MPS was observed in the first season after ACLR, while values in the second and third seasons were similar to the preinjury period	NA	NA	NA
Forsythe et al. (2021) [38]	GPS, MPS, MPG, GLPS, APS, and PPG noticeably declined relative to the season before the injury	No differences were observed in goalkeepers, defenders, and midfielders in relevant outcomes compared with their healthy peers. Only attackers’ performances have deteriorated	NA	NA
Mazza et al. (2022) [41]	The mean preoperative MPS decreased in the first, second, and third seasons after ACLR	NA	13.6% of soccer players ended their careers within 3 seasons after ACLR	NA
Niederer et al. (2018) [42]	MPS, tackles per game, and the number of completed passes decreased over time	A statistically significant group x time interaction was recorded in the variables of scoring points, number of completed passes, dribbling, and MPS in favor of the control group	NA	ACLR group athletes had significantly shorter careers compared with their healthy counterparts
Sandon et al. (2020) [43]	NA	NA	4.9 years after ACLR	NA
Szymski et al. (2023) [45]	No differences were noted in MPS in elite soccer players 2 seasons after ACLR compared with the period before the injury. On the other hand, MPS significantly decreased among semi-professional and amateur athletes	NA	A third of athletes ended their careers 3 seasons after ACLR	NA

*ACLR* anterior cruciate ligament reconstruction, *APS* assists per season, *GCPS* goals conceded per season, *GLPS* goals per season, *GPS* games played per season, *MPG* minutes played per game, *MPS* minutes played per season, *NA* not applicable, *PPG* points per game, *SGPS* shot on goal per season, *SPS* shots per season

Six papers [30, 32, 35, 41, 43, 45] addressed the career length of soccer players following ACLR. The mean career duration of athletes after ACL surgery was between 4 and 5 years (Table 3). Two articles [30, 42] provided data in terms of a comparison of career length between the ACLR group and their healthy counterparts. In both studies, soccer players that underwent ACLR had significantly shorter careers relative to the control group of athletes.

### Quality Assessment

There were 13 noncomparative studies [29, 31–35, 37, 39–41, 43–45] and four studies [30, 36, 38, 42] with a control group (Supplementary material 1, see electronic supplementary material [ESM]). In investigations without a control group, quality scores ranged between 8 and 13, with an overall mean quality score of 10.8. Therefore, the quality of noncomparative studies can be defined as moderate. Similarly, the range of quality scores for comparative studies was between 15 and 21, and the overall mean quality score was 17.8; hence, the quality of articles that involved a control group of athletes was also moderate. The most critical items were the unbiased assessment of the study endpoints, the aspect that referred to the loss to follow-up, and the calculation of the sample size necessary for the research.

## Discussion

This systematic literature review aimed to summarize the scientific evidence available over the last 5 years regarding RTP and performance in soccer players after ACLR. The results obtained demonstrated that 72% of athletes RTP following surgery, while 53% of them successfully RTP at the preinjury level of participation. Concerning the time necessary for RTP, the literature indicated that 264 days or 8.7 months was the average period between ACLR and the first soccer match appearance. Moreover, a marked decline in performance was observed in terms of both comparisons with the season before ACLR and with the control group of soccer players. Finally, the mean career duration of athletes following ACLR was approximately 4–5 years.

### RTP After ACLR in Soccer Players

The findings related to RTP and RTP at the preinjury level are partially in agreement with the currently available evidence. More specifically, a literature review that included exclusively NFL players demonstrated that a total of 67.2% of athletes RTP following primary ACL surgery [25]. Additionally, DeFazio et al. [46] revealed that 73.2% of athletes that mainly competed in soccer, basketball, and the NFL successfully RTP after ACLR. In line with the presented findings, RTP at the preinjury level of competition in pivoting sports was 51% [47] and 53% [48]. On the other hand, several studies highlighted a significantly higher percentage of athlete RTP and RTP at the preinjury level relative to the results obtained in this research. Namely, 83% [18] and between 78% and 86% [24] of elite athletes and NBA players, respectively, RTP following ACL surgery. In handball, it was reported that 58% of athletes from the three upper Norwegian divisions RTP at the preinjury level of sport participation [49]. Several factors likely contributed to the inconsistency between the highlighted evidence and the results obtained in this study. More precisely, although the articles most commonly involved elite soccer players, it is obvious that a substantially lower percentage of athletes RTP and RTP at the preinjury level in investigations that comprised national-level, amateur, or recreational athletes. For example, Sandon et al. [43] revealed that 51% of national, recreational, and youth soccer players RTP following ACLR. It is also relevant to emphasize that impaired values of the examined parameters were observed in studies that included female soccer players. Specifically, it was demonstrated that only 37% of female athletes RTP at the preinjury level of competition after ACL surgery [34]. Overall, lower levels of competition and female sex are considered factors that potentially negatively affect RTP and RTP at the preinjury level of participation. Nonetheless, future studies are warranted to more clearly understand their influence on the mentioned variables in soccer players that underwent ACLR.

The presented literature review indicated that the average RTP time after ACLR was 8.7 months. These findings are in line with the recently conducted systematic review and meta-analysis by Hong et al. [50]. The authors reported that the soccer players were able to RTP following a period of between 6.1 and 11.1 months. However, even though 8.7 months represents a significant period of absence from soccer matches, the recovery time highlighted in this research is considerably shorter compared with studies performed on samples of NFL [51, 52], NBA [24], school-aged [53], amateur [54], and recreational athletes [55]. For instance, RTP times of 9.8 [24] and 12.6 [52] months were recorded for NBA and NFL players, respectively. In addition, RTP at the preinjury level of competition in recreational athletes was 33.7 months [55]. Overall, it should also be noted that prolonged RTP time was found in studies that comprised recreational and amateur soccer players [40, 45]. Moreover, based on the results obtained in this research, it appears that the revision of ACL surgery detrimentally influenced the RTP time of the examined population. Furthermore, as well as in this study, there is convincing evidence in the scientific literature that fear of reinjury [56–58] and knee-related issues [48, 59] were the most cited reasons for not returning to sport. Therefore, since fear of repeating an ACL injury was likely the major cause for quitting soccer, certain psychological interventions could be helpful to overcome this issue. In other words, cooperation among physiotherapists, soccer coaches, and sports psychologists appears indispensable to increase the number of soccer players that RTP after ACLR and to improve the health of their knees.

### Performance and Career Length After ACLR in Soccer Players

In terms of summarizing the literature, several studies examined the performance of athletes after ACLR [18, 25, 60]. For example, Mohtadi and Chan [60] reported a deterioration in athletes’ performance that referred to the statistical aspects following ACL surgery. Likewise, the performance of NFL players that underwent primary ACLR significantly declined compared with a noninjured control group [25]. In contrast, after ACLR, no deterioration in performance was observed in a systematic review with meta-analysis that evaluated exclusively elite athletes [18]. Nevertheless, the findings related to performance in the presented research were quite unambiguous. As previously emphasized, most of the statistical parameters decreased in the seasons after ACLR relative to the preinjury period. Moreover, two of the three studies demonstrated a decline in performance in the ACLR group of soccer players compared with their healthy colleagues. Therefore, taking into account the importance of statistics in modern soccer in terms of distinguishing more successful athletes from less successful ones, there is an obvious need for prevention and rehabilitation programs to reduce the adverse effects of ACL injuries in soccer players, irrespective of their age, sex, or level of competition.

As in the case of performance, the career length of soccer players that underwent ACL surgery was noticeably shorter relative to noninjured athletes. These findings are supported by the available literature. Specifically, NFL wide receivers ended their professional careers approximately 1.9 seasons earlier compared with their matched controls [19]. In addition, women NBA athletes with a history of ACLR had significantly shorter sports careers than their noninjured counterparts [61]. Furthermore, according to the recent scientific evidence provided in this study, the mean career length of soccer players after ACLR was between 4 and 5 years. Consistent with the presented results, several investigations also reported that the mean career duration of NFL players following ACLR was 4.8 years [62], and 4.5 years in hockey players [23]. Overall, based on the highlighted facts, it can be inferred that ACL injuries negatively impacted the career duration of soccer players. Similarly, as with performance, a collaboration between physiotherapists and soccer coaches seems necessary to preserve athletes’ health and extend their careers.

### Strengths, Limitations, and Recommendations for the Future Studies

There are certain strengths of this systematic review that must be emphasized. Primarily, all included studies have been published within the last 5 years, thus providing very innovative evidence relating to the RTP and performance of soccer players after ACLR. Moreover, more than half of the investigations addressed only elite soccer players competing in the most prestigious European soccer leagues. Nonetheless, despite this fact, to amplify the currently available knowledge, it is recommended that several studies be conducted in top soccer leagues in other continents, including South America (e.g. Brazil and Argentina). Finally, concerning the practical implications of the research, the results obtained are truly useful for physiotherapists and soccer coaches who are engaged in professional soccer.

Conversely, some apparent limitations should be acknowledged. The absence of a control group was observed in several studies that evaluated the performance and career length of soccer players. Additionally, moderate quality was recorded in both comparative and noncomparative investigations. Therefore, future studies with higher methodological quality, including the presence of a control group, are warranted to verify the presented findings. Furthermore, only two articles assessed the examined variables in soccer players that were 18 years old or younger. Most importantly, future studies need to evaluate RTP and performance in youth soccer athletes that experienced ACL rupture. The majority of the included studies encompassed a sample of athletes that underwent both primary and revision ACL surgery, which potentially negatively affected outcomes estimated, particularly the performance and career length of soccer players.

## Conclusion

The main findings of this literature review indicate that 72% of soccer players RTP following ACLR and 53% RTP at the preinjury level of competition, with an RTP time of 8.7 months. However, although a high percentage of athletes successfully returned to soccer after ACLR with a relatively short period of absence from the soccer field compared with sports closely related to soccer, performance and career duration markedly deteriorated relative to the control group. Hence, physiotherapists, coaches, and soccer players themselves must be aware of the detrimental effects of ACL rupture, and they need to try to create efficient prevention and rehabilitation programs that would completely neutralize or reduce the consequences induced by this injury.

### Supplementary Information

Below is the link to the electronic supplementary material.

Supplementary file1 (PDF 143 KB)

## Declarations

### Availability of Data and Material

Considering that this study is a systematic review of literature, all the necessary data are available in the manuscript and supplementary material.
