# Supplementary material for: Return to Play and Performance After Anterior Cruciate Ligament Reconstruction in Soccer Players: A Systematic Review of Recent Evidence
Source: Sports Med. 2024 May 6;54(8):2097–108. doi: 10.1007/s40279-024-02035-y (PMC11329701; doi:10.1007/s40279-024-02035-y)
Supplement: Supplementary file 1 — Supplementary file1 (PDF 143 KB) [file 40279_2024_2035_MOESM1_ESM.pdf]

Supplementary material

Return to Play and Performance After Anterior Cruciate Ligament Reconstruction in Soccer Players: A Systematic Review of Recent Evidence

Marko Manojlovic[1*], Srdjan Ninkovic[2,3], Radenko Matic[1], Sime Versic[4], Toni Modric[4], Damir Sekulic[4] Patrik Drid[1]

[1] Faculty of Sport and Physical Education, University of Novi Sad, Novi Sad, Serbia
[2] Faculty of Medicine, University of Novi Sad, Novi Sad, Serbia
[3] Department of Orthopedic Surgery and Traumatology, Clinical Center of Vojvodina, Novi Sad, Serbia
[4] Faculty of Kinesiology, University of Split, Split, Croatia

Corresponding author:
Marko Manojlovic
Faculty of Sport and Physical Education
University of Novi Sad, Novi Sad, Serbia
Email: markomanojlovic1995@gmail.com

Supplementary material 1 Quality assessment with MINORS

Author (year)	1	2	3	4	5	6	7	8	9	10	11	12	Total score
Alonso et al. (2019) [29]	2	2	2	1	0	2	1	1	NA	NA	NA	NA	11/16
Arundale et al. (2018) [30]	2	2	2	2	1	2	0	2	2	2	2	2	21/24
Balendra et al. (2022) [31]	2	1	2	2	0	2	2	0	NA	NA	NA	NA	11/16
Barth et al. (2019) [32]	1	2	2	1	0	2	0	0	NA	NA	NA	NA	8/16
Bonanzinga et al. (2022) [33]	2	2	2	1	0	2	1	1	NA	NA	NA	NA	11/16
Britt et al. (2020) [34]	2	2	1	1	0	2	1	1	NA	NA	NA	NA	10/16
Della Villa et al. (2021) [35]	2	1	2	2	1	2	2	1	NA	NA	NA	NA	13/16
Fältström et al. (2021) [36]	2	2	2	1	0	2	2	1	1	2	2	2	19/24
Farinelli et al. (2023) [37]	2	2	2	1	0	2	2	1	NA	NA	NA	NA	12/16
Forsythe et al. (2021) [38]	1	2	2	1	0	2	0	1	2	2	2	1	16/24
Manara et al. (2022) [39]	2	2	2	1	1	2	1	1	NA	NA	NA	NA	12/16
Mars Group (2021) [40]	2	1	2	1	0	2	1	1	NA	NA	NA	NA	10/16
Mazza et al. (2022) [41]	2	1	2	2	0	2	1	1	NA	NA	NA	NA	11/16
Niederer et al. (2018) [42]	2	1	2	2	0	2	1	0	2	1	0	2	15/24
Sandon et al. (2020) [43]	2	1	2	1	0	2	1	1	NA	NA	NA	NA	10/16
Schiffner et al. (2018) [44]	2	1	2	2	1	2	0	1	NA	NA	NA	NA	11/16
Szymski et al. (2023) [45]	2	2	1	1	1	2	1	1	NA	NA	NA	NA	11/16

Note: MINORS - methodological index for non-randomized studies; NA – not applicable. All items are scored 0 (not reported), 1 (reported but inadequate), and 2 (reported and adequate). The maximum score for non-comparative studies is 16 and the maximum score for comparative studies is 24. 1 - a clearly stated aim; 2 - inclusion of consecutive patients; 3 - prospective collection of data; 4 - endpoints appropriate to the aim of the study; 5 - unbiased assessment of the study endpoint; 6 - follow-up period appropriate to the aim of the study; 7 - loss to follow up less than 5%; 8 - prospective calculation of the study size; 9 - an adequate control group; 10 - contemporary groups; 11 - baseline equivalence of groups; 12 - adequate statistical analyses.
